# Research progress on extracellular vesicles in the renal tubular injury of diabetic kidney disease

**DOI:** 10.3389/fendo.2023.1257430

**Published:** 2023-09-04

**Authors:** Shengjie Li, Shanshan Zheng, Jiao Li, Sen Lin, Hao Li, Ping Wang, Ping Chen, Chaoqun Ma, Yipeng Liu

**Affiliations:** ^1^ Department of Nephrology, The First Affiliated Hospital of Shandong First Medical University & Shandong Provincial Qianfoshan Hospital, Jinan, China; ^2^ Department of Nephrology, Binzhou People’s Hospital Affiliated to Shandong First Medical University, Binzhou, China; ^3^ Department of Nephrology, Shandong Provincial Qianfoshan Hospital, Shandong University, Jinan, China; ^4^ Nephrology Research Institute of Shandong Province, Jinan, China; ^5^ Department of Emergency Medicine, Shandong Provincial Hospital Affiliated to Shandong First Medical University, Jinan, China

**Keywords:** diabetic kidney disease, renal tubular injury, extracellular vesicles, exosomes, miRNA

## Abstract

Diabetic kidney disease (DKD) is a severe microvascular complication of diabetes and is a chronic progressive condition. It is also a common cause of end-stage renal disease (ESRD), which is characterized by proteinuria or a progressive decline in the glomerular filtration rate. Due to their dependence on high-energy and aerobic metabolism, renal tubules are more susceptible to the metabolic disturbances associated with DKD, leading to inflammation and fibrosis. Consequently, tubular injury has become a recent research focus, and significant advancements have been made in studying the role of extracellular vesicles in DKD-associated tubular injury. This review aimed to elucidate the mechanisms and potential applications of different types of extracellular vesicles in tubular injury in DKD to provide new insights for the prevention and treatment of DKD.

## 1 Introduction

Diabetic kidney disease (DKD) is the most severe microvascular complication of diabetes and a common cause of end-stage renal disease (ESRD). As a chronic progressive disease, DKD ranks first among the etiologies of chronic kidney disease (CKD) in developed countries and is a major reason for renal replacement therapy worldwide (1). Prolonged hyperglycemia in DKD patients damage and disrupt glomerular and tubular cell structures, as well as the microvascular system. This, in turn, induces pathological changes such as progressive thickening of the glomerular basement membrane, mesangial matrix expansion, glomerular hyperfiltration, podocyte injury, extracellular matrix deposition, and tubulointerstitial fibrosis (1, 2). Clinically, as DKD progresses, it is primarily characterized by worsening proteinuria, which is closely associated with the incidence and mortality of cardiovascular complications. Therefore, current treatment approaches for DKD primarily focus on strict control of blood glucose and blood pressure, as well as blockade of the renin-angiotensin-aldosterone system to reduce proteinuria and the occurrence of cardiovascular diseases. However, effective strategies to halt or reverse the progression of DKD are still lacking (3, 4). Therefore, a comprehensive understanding of the pathogenesis of DKD may provide new research directions for the treatment of this disease.

The pathogenesis of DKD is complex and not yet fully understood. It is widely accepted that podocyte injury is a central factor in the development of DKD (5). However, with further research, tubular injury has gradually emerged as a recent focus. Renal tubules are epithelial tubules connected to the renal capsule that play a critical role in reabsorption, the excretion of metabolic waste products, and the maintenance of fluid and acid-base balance in the body. Tubular epithelial cells (TECs) are the most abundant cell type in the kidney and have important regulatory functions under normal and pathological conditions. Due to their dependence on high-energy and aerobic metabolism, tubules are more susceptible to the metabolic disturbances associated with diabetes, leading to oxidative stress, the secretion of various cytokines, and the occurrence of interstitial inflammation and fibrosis (6). This makes tubules a vulnerable site for DKD-associated renal injury. TECs, which are at the center of renal damage, are also highly susceptible to injury and destruction in DKD. In response to high glucose and proteinuria, damaged TECs can secrete various profibrotic and proinflammatory cytokines through autocrine or paracrine mechanisms, inducing cell detachment and apoptosis and thereby exacerbating renal fibrosis and promoting the progression of DKD (7, 8). Similarly, research has indicated that tubular injury may be the primary cause of renal function decline or renal failure in DKD patients (6, 9). These findings suggest that tubular injury is a crucial component of DKD pathophysiology, and therapeutic interventions targeting tubules may become a key focus in future disease prevention and treatment efforts.

Extracellular vesicles (EVs) are heterogeneous membrane-bound vesicles released by cells and include exosomes, microvesicles, apoptotic bodies, and oncosomes. EVs play a role in mediating intercellular communication and signal transduction. EVs can be secreted by various renal cells and participate in the pathophysiological mechanisms of DKD through intercellular crosstalk induced by high glucose. EVs also have the potential to serve as biomarkers for disease diagnosis (10, 11). Currently, the gold standard for diagnosing DKD is renal biopsy, and there is a lack of noninvasive early diagnostic indicators with high sensitivity and specificity. Therefore, EVs, which are noninvasive biomarkers involved in the progression of DKD, have gained widespread attention.

## 2 Biological characteristics of extracellular vesicles

EVs are heterogeneous membrane-bound vesicles released by various cells into the extracellular environment. Based on their release mechanisms, size, and the expression of biomarkers, EVs can be categorized as exosomes, microvesicles, apoptotic bodies, and oncosomes, and exosomes and microvesicles are the two major types (11, 12). Initially, EV secretion was thought to be a way for cells to clear metabolic waste products. However, subsequent research revealed that EVs play important roles in intercellular communication by transferring signals to neighboring or distant target cells through processes such as endocytosis, phagocytosis, and membrane fusion, thereby maintaining cellular homeostasis (13). Studies have shown that EVs contain various bioactive substances derived from parent cells, including proteins, carbohydrates, lipids, and various genetic materials (DNA, mRNA, miRNA, and other noncoding RNAs), and they play important roles in intercellular communication and signal transduction by delivering these substances to recipient cells (14).

Exosomes are nanoscale extracellular vesicles with diameters of approximately 30-100 nm that are released by cells under different stress conditions. Their biogenesis is a continuous process involving the inward budding of the plasma membrane and the formation of intraluminal vesicles within intracellular multivesicular bodies (MVBs). These mature MVBs can either fuse with lysosomes or autophagosomes for degradation or fuse with the plasma membrane and release their intraluminal vesicles as exosomes into the extracellular space. These exosomes can then be taken up by recipient cells and mediate intercellular communication through various mechanisms (15). Almost all exosomes have a set of evolutionarily conserved proteins, including tetraspanins (CD9, CD63, and CD81), Alix, and TSG101, which serve as biomarkers for exosome identification. They also contain unique proteins that reflect their cellular origins or cell type specificity. Furthermore, exosomes have been shown to be secreted and taken up by cells of all types in the human body. They are widely present in various body fluids, such as plasma, urine, saliva, cerebrospinal fluid, breast milk, amniotic fluid, semen, and pleural or peritoneal effusions and carry a variety of proteins, lipids, and genetic materials (DNA, mRNA, miRNA, and other noncoding RNAs) as bioactive substances involved in the biological regulation of target cells (16).

Microvesicles (MVs) are small vesicles formed by the shedding of the plasma membrane from healthy, activated, or apoptotic cells and have diameters of approximately 50-2000 nm. MVs and exosomes differ significantly in their biogenesis and membrane surface biomarkers (12). On the one hand, MVs are formed through direct budding and fission of the plasma membrane, which is closely related to the disruption of membrane asymmetry and the degradation of cytoskeletal proteins (17). On the other hand, MVs are typically identified by biomarkers such as integrins, glycoprotein Ib (GPIb), P-selectin, and phosphatidylserine, which are often not detected in exosomes. Similar to exosomes, MVs are widely present in various body fluids and tissues and contain and transport various bioactive substances. They interact with adjacent or distant target cells through multiple mechanisms, participate in communication between cells and play important roles.

MicroRNAs (miRNAs) are endogenous, short noncoding small RNA molecules with lengths of approximately 18-22 nucleotides. In cells, miRNAs can induce mRNA degradation or inhibit protein synthesis by binding to the 3’ untranslated region (3’UTR) of mRNA, thereby negatively regulating the expression of target genes (18). As a type of noncoding RNA, miRNAs are highly conserved and exhibit spatiotemporal specificity, making them a research hotspot in recent years. Moreover, with further research, increasing evidence suggests that dysregulated miRNAs are involved in the occurrence and development of kidney diseases and are closely associated with processes such as inflammation, fibrosis, and epithelial-mesenchymal transition (19, 20).

## 3 Potential diagnostic value of exosomes in tubular injury in DKD

Currently, the main clinical manifestations of DKD include persistent albuminuria and/or a reduction in the estimated glomerular filtration rate (eGFR). The urinary albumin-to-creatinine ratio (UACR) is widely used as a cornerstone for diagnosing DKD in clinical practice, and significant levels of macroalbuminuria (UACR > 300 mg/g) or microalbuminuria (UACR 30-300 mg/g) are routine biomarkers for the early diagnosis of DKD (21). However, recent research has revealed certain differences between the clinical manifestations of DKD in patients and the severity of renal damage. Some patients may already have advanced glomerular lesions even when exhibiting microalbuminuria or no proteinuria (4), indicating a lack of sensitivity and specificity of proteinuria as a biomarker for disease diagnosis. Therefore, it is critical to identify new biomarkers associated with disease diagnosis. With further research on exosomes, it has been discovered that exosomes, which are important mediators of intercellular communication, have extensive application prospects in the diagnosis of DKD. In 2014, Lv et al. (22) reported that compared to that in the healthy control group, the mRNA expression of CD2AP was downregulated in DKD patients and closely correlated with renal function, proteinuria levels, and the degree of tubulointerstitial fibrosis. Therefore, the expression of urinary exosomal CD2AP mRNA can serve as a noninvasive tool for monitoring renal function and the degree of renal fibrosis in DKD patients, providing new possibilities for disease diagnosis. In 2018, Yu et al. (23) conducted a clinical data study and found that compared to that in healthy controls, urinary exosomal miR-200b was reduced in DKD patients with progression of tubulointerstitial fibrosis, and the reduction was more significant in exosomes derived from nonproximal tubules. Consequently, nonproximal tubule-derived urinary exosomal miR-200b can be used as a biomarker to predict the degree of renal fibrosis in DKD, replacing traditional invasive renal biopsy as a novel tool for diagnosing tubular injury in DKD. In 2020, Ning et al. (24) evaluated the diagnostic value of α1-antitrypsin (α1-AT) in urinary exosomes at different stages of diabetes. They found that the expression of α1-AT was not detected in urinary exosomes from healthy individuals and prediabetic patients but was significantly upregulated in urinary exosomes from normoalbuminuric diabetic patients, and the expression level increased with the occurrence and progression of proteinuria. *In vitro* cell experiments demonstrated increased expression of α1-AT in HK-2 cells under high glucose conditions, which was accompanied by increased expression of the inflammatory factors MCP-1 and TNF-α, indicating that α1-AT has proinflammatory effects on DKD. These studies suggest that α1-AT expression in urinary exosomes may serve as a noninvasive biomarker for the early diagnosis of DKD and predict the decline in renal function to some extent.

## 4 Different origins of exosomes and tubular injury

Studies have shown that various renal cells, including podocytes, renal tubular epithelial cells, glomerular endothelial cells, and mesangial cells, can secrete exosomes (25). Under stress conditions such as hypoxia, acidic pH, high glucose, oxidative stress, and uremic toxins, the quantity and content of exosomes can change (26–29). Moreover, exosomes can induce the release of cytokines and promote the accumulation of inflammatory cells, and exosomes secreted by damaged renal cells can also be transferred to normal renal tissue, altering their phenotype and inducing interactions between cells. Therefore, as important mediators of intercellular communication, the role of exosomes from different sources in tubular injury in diabetic kidney disease (DKD) has become a recent research focus.

### 4.1 Renal tubular epithelial cell-derived exosomes and tubular injury

Diabetic kidney disease (DKD) is typically regarded as a glomerular disease, but changes in the tubulointerstitium also play a crucial role in disease progression. Tubular injury and dysfunction in tubular reabsorption are important factors in the development of proteinuria. Recent studies have shown that autologous exosomes have significant implications in mediating intercellular crosstalk among tubular cells. Tsai et al. (30) showed that exosomes derived from proximal tubular epithelial cells (PTECs) mediated tubular cell intercommunication through the miR-1269b/FBLN1 pathway, thus inducing tubulointerstitial fibrosis in DKD. *In vitro* experiments confirmed the downregulation of miR-1269b expression in HK-2 cells under high glucose conditions and the regulation of FBLN1 expression in PTEC-derived exosomes, which resulted in increased FBLN1 expression and the subsequent induction of epithelial-mesenchymal transition (EMT) in PTECs. Furthermore, increased levels of FBLN1 and decreased levels of miR-1269b were observed in urine exosomes from a T2DM mouse model and patients, and the levels of FBLN1 and miR-1269b in urine exosomes correlated with the severity of tubular injury in T2DM patients. The proposed regulatory mechanism of miR-1269b/FBLN1 may offer new insights for the treatment of DKD patients in clinical practice. In another study by Wen et al. (31), it was reported that DKD patients had decreased secretion of tubular cell-derived exosomes. *In vitro* experiments demonstrated that exosomes derived from HK-2 cells under high glucose conditions could stimulate fibroblast proliferation. Additionally, proteomic analysis of exosomes revealed the involvement of Eno1 in this process, providing evidence for cell communication mediated by tubular-derived exosomes in DKD renal fibrosis. Li et al. (9) noted that CHAC1 expression was significantly increased in inflammation-related diseases, and the NF-κB signaling pathway played a critical role in various types of renal injury and was associated with CHAC1 in inflammation. Subsequently, cell experiments confirmed that inhibiting the secretion of exosomal miR-26a-5p from BSA-induced HK-2 cells suppressed the CHAC1/NF-κB pathway and inhibited the inflammatory response in HK-2 cells, thus delaying the progression of DKD. This study provides new insights into the protective role and pathogenic mechanisms of PTEC-derived exosomes in DKD. Moreover, the identification of the miR-26a-5p/CHAC1/NF-κB pathway also presented a new therapeutic target for DKD.

### 4.2 Podocyte-derived exosomes and tubular injury

Podocytes are terminally differentiated cells located on the outer side of the glomerular basement membrane that contribute to the formation of the glomerular filtration barrier along with the basement membrane and endothelial cells. Under pathological conditions in diabetic kidney disease (DKD), any harmful factors such as high blood glucose, an increase in advanced glycation end-products, oxidative stress, and inflammation can result in permanent damage to podocytes. This disruption of filtration barrier integrity leads to proteinuria, and proteinuria itself induces tubulointerstitial fibrosis (32). In addition, since podocytes can release exosomes and are located adjacent to the proximal tubule, Lv et al. (33) suggested in 2019 that the proximal tubule could be a site for interactions of exosomes in podocytes. In 2020, Jeon et al. (34) reported that miR-424 was upregulated in damaged podocyte-derived exosomes, which induced fibrosis or apoptosis in renal tubular epithelial cells by activating the p38 signaling pathway, confirming that podocyte-derived exosomes could mediate cell communication between podocytes and the proximal tubule. In the same year, Huang et al. (35) extracted exosomes from podocytes cultured under high glucose, normal glucose, and iso-osmotic conditions and cocultured them with proximal tubular epithelial cells (PTECs). They showed for the first time that exosomes secreted by podocytes under high glucose conditions induced apoptosis in PTECs and identified 5 differentially expressed miRNAs in podocytes through miRNA sequencing, providing new insights for the diagnosis and treatment of diabetic kidney disease. Similarly, Su et al. (36) found that under high glucose conditions, podocyte-derived exosomes carrying miR-221 activated the Wnt/β-catenin signaling pathway in PTECs by targeting DKK2, resulting in proximal tubular cell injury. These studies collectively demonstrate the crucial role of podocyte-derived exosomes in proximal tubular cell injury in diabetes, providing new insights into the mechanisms underlying proximal tubular cell injury in diabetic kidney disease and bearing important clinical predictive and guiding significance.

### 4.3 Macrophage-derived exosomes and tubular injury

Diabetic kidney disease (DKD) is an inflammatory disease caused by metabolic disorders, and toxic lipid-induced apoptosis in tubular epithelial cells and the infiltration of inflammatory cells are typical pathological features of disease progression. Macrophage infiltration plays an important role in regulating innate and adaptive immune responses in DKD. Chow et al. (37) reported macrophage infiltration in the kidney tissues of diabetic mice. The number of macrophages increased with disease duration and was closely associated with the severity of kidney injury and fibrosis. However, the defense mechanisms and cell signaling pathways used by infiltrating macrophages in DKD have not been clearly elucidated. Subsequently, Jiang et al. (38) discovered a novel cell communication mechanism between tubular epithelial cells and macrophages in a type 2 diabetic mouse model. The study demonstrated that exosomes secreted by DKD tubular epithelial cells, which are rich in LRG1, activate the inflammatory phenotype of macrophages through the TGF-β-R1 pathway and induce the release of exosomes from macrophages. Furthermore, the LRG1/TGF-βR1 signaling pathway increased the expression of TRAIL in macrophages, and exosomes derived from TRAIL-rich macrophages further induced apoptosis in tubular epithelial cells, thus exacerbating kidney inflammation and injury. These findings suggest that the mechanisms of cell communication between macrophages and tubular epithelial cells play a crucial role in tubular injury in DKD, and targeting the inflammatory pathway may be a crucial step in preventing and controlling disease progression and may provide a new drug target for the treatment of DKD.

### 4.4 Urine-derived exosomes and tubular injury

Previous studies have suggested that miRNAs are important mediators of the development of DKD and are closely associated with tubulointerstitial fibrosis, serving as potential biomarkers for evaluating disease progression (19, 20). Urine-derived exosomes are extracellular vesicles released from the urogenital tract that reflect the degree of kidney damage. Similar to other types of exosomes, urine-derived exosomes contain miRNAs, and compared to free miRNAs, miRNAs in urine-derived exosomes are more stable and less affected by endogenous RNAase activity, making them suitable as biomarkers for assessing tubular injury (39). For instance, in 2013, Lv et al. (40) showed that the expression of miR-29c in urine-derived exosomes was increased in CKD patients, including those with DKD, compared to healthy controls and was closely correlated with the degree of renal fibrosis. This finding suggests that miR-29c can serve as a novel noninvasive biomarker for renal fibrosis, providing a new approach to disease diagnosis. In 2016, Jiang et al. (41) discovered a potential therapeutic application of urine-derived exosomes in DKD. They found that intravenous injection of urine-derived stem cell-secreted exosomes (USC-Exos) reduced urine volume and urinary microalbumin excretion and inhibited excessive caspase-3 expression in diabetic rats, thereby suppressing apoptosis in tubular epithelial cells and podocytes and promoting proliferation of glomerular endothelial cells. Therefore, the application of USC-Exos may provide a new therapeutic option for DKD treatment. Jia et al. (42) reported in 2018 that miR-4756 expression was increased in urine-derived exosomes from DKD patients compared to those from patients with simple diabetes. Subsequent cellular experiments further confirmed that the increase in miR-4756 induced epithelial-mesenchymal transition and endoplasmic reticulum stress in HK-2 cells cultured under high-glucose conditions by targeting Sestrin2, thereby promoting tubulointerstitial fibrosis and DKD progression. Overall, urine-derived exosomes, which are derived from renal tissue, have inherent advantages, such as simple and noninvasive extraction methods, making them more promising for the diagnosis and treatment of DKD than other types of exosomes.

## 5 Microvesicles and tubular injury

In addition to exosomes, microvesicles, which are extracellular vesicles capable of carrying various bioactive components (such as lipids, cytokines, DNA, RNA, miRNAs, and other noncoding RNAs) and mediating cell-to-cell communication, have also become a research focus in recent years. Studies have indicated that microvesicles play an important role in cellular communication in DKD and are involved in disease progression. Burger et al. (43) found in 2014 that the secretion of microvesicles by podocytes was increased in diabetic mice compared to nondiabetic mice, suggesting that microvesicles derived from glomerular podocytes could serve as early urine biomarkers for podocyte injury in DKD. Furthermore, in 2018, Munkonda et al. (44) confirmed that microvesicles were closely associated with tubular epithelial cell injury. Their study revealed that microvesicles derived from human podocytes stimulated fibrotic signaling in human proximal tubular epithelial cells when the cells were cocultured under high-glucose conditions. In 2019, Ravindran et al. (45) reported that microvesicles released by proximal tubular epithelial cells under high-glucose conditions activated multiple pathways, including the mTOR, ERK, TGF-β, and EMT pathways, in neighboring tubular epithelial cells. These pathways induce tubulointerstitial inflammation and fibrosis, exacerbating tubular injury and promoting DKD progression. This finding contributes to a better understanding of the mechanisms by which microvesicles induce tubular epithelial cell injury in DKD and provide new biological targets for the diagnosis and treatment of DKD.

## 6 Conclusion

Despite significant progress in the treatment of DKD in recent years, this disease remains the leading cause of end-stage renal disease. Early diagnosis and timely intervention can greatly delay disease progression, and therefore, research on emerging biomarkers associated with DKD diagnosis, including circulating miRNAs and extracellular vesicles, is ongoing. Various experimental therapies, such as stem cell treatments, are also being explored. Studies have shown that the injection of extracellular vesicles derived from bone marrow mesenchymal stem cells into the kidneys of diabetic animal models can reduce tubular inflammatory cell infiltration, improve renal histopathological damage, and effectively alleviate renal fibrosis (46), providing new research strategies for DKD treatment. This article primarily discussed the pathological and physiological mechanisms, diagnosis, and therapeutic prospects of different types of extracellular vesicles associated with tubular injury in DKD. Exosomes from different sources (such as macrophages, podocytes, tubular epithelial cells, and urine) and microvesicles mediate DKD progression through the activation of various inflammatory pathways, thereby contributing to a better understanding of the mechanisms underlying tubular injury in DKD and providing new biological targets for the diagnosis and treatment of this disease (**Table 1**). However, most current research is still limited to cellular and animal experiments, and clinical studies with larger cohorts are needed to further validate the role of extracellular vesicles in tubular injury in diabetic kidney disease.

**Table 1:** Potential diagnostic value of exosomes from different sources in DKD.

The origin of exosomes	The contents of exosomes	Level compared with healthy individuals	Type of damage
**Urine-derived Exosomes**	FBLN1 and α1-antitrypsin	Increased	Tubular epithelial cell injury
	miR-1269b	Decreased	
	CD2AP mRNA	Decreased	Fibrosis
	miR-200b	Decreased	
	miR-29c and miR-4756	Increased	
**Renal Tubular Epithelial Cell-derived Exosomes**	FBLN1	Increased	Tubular epithelial cell injury
	miR-1269b	Decreased	

## Author contributions

SL: Writing – original draft, Writing – review and editing. SZ: Writing – review and editing. JL: Writing – review and editing. SL: Writing – review and editing. HL: Writing – review and editing. PW: Writing – review and editing. PC: Writing – review and editing. CM: Writing – review and editing. YL: Writing – review and editing, Conceptualization.
